# Adaptive divergence along environmental gradients in a climate-change-sensitive mammal

**DOI:** 10.1002/ece3.776

**Published:** 2013-09-16

**Authors:** P Henry, M A Russello

**Affiliations:** Department of Biology, University of British ColumbiaOkanagan Campus, 3333 University Way, Kelowna, BC, V1V 1V7, Canada

**Keywords:** Adaptation, climate change, conservation genetics, *Ochotona princeps*, population genetics – empirical

## Abstract

In the face of predicted climate change, a broader understanding of biotic responses to varying environments has become increasingly important within the context of biodiversity conservation. Local adaptation is one potential option, yet remarkably few studies have harnessed genomic tools to evaluate the efficacy of this response within natural populations. Here, we show evidence of selection driving divergence of a climate-change-sensitive mammal, the American pika (*Ochotona princeps*), distributed along elevation gradients at its northern range margin in the Coast Mountains of British Columbia (BC), Canada. We employed amplified-fragment-length-polymorphism-based genomic scans to conduct genomewide searches for candidate loci among populations inhabiting varying environments from sea level to 1500 m. Using several independent approaches to outlier locus detection, we identified 68 candidate loci putatively under selection (out of a total 1509 screened), 15 of which displayed significant associations with environmental variables including annual precipitation and maximum summer temperature. These candidate loci may represent important targets for predicting pika responses to climate change and informing novel approaches to wildlife conservation in a changing world.

## Introduction

A significant challenge facing wildlife species will be coping with contemporary climate change. When subject to environmental stresses, species have three options: disperse, adapt, or go extinct (Hewitt and Nichols 2005). While it has been suggested that most species will shift their geographical ranges rather than adapt *in situ* (Parmesan 2006), factors such as habitat fragmentation may act synergistically to impair species' dispersal to more favorable conditions. Distributional shifts may be particularly challenging for species with limited dispersal capacity or those with highly restricted habitat requirements; thus, local adaptation may represent the only option for continued persistence (Sgrò et al. 2011).

The identification of genes underlying ecologically important traits has traditionally been limited to well-studied or model organisms using a targeted gene approach within systems where individual adaptive traits segregate into contrasting phenotypes (e.g., detection of the genetic basis of fur coloration in mammals; Hoekstra 2006). Additionally, associations between phenotypes and genotypes have been characterized using quantitative trait loci, yet such studies are restricted to well-studied taxa that can be experimentally manipulated and crossed. Moving beyond model systems, the development of population genomic approaches (Luikart et al. 2003) has enabled the investigation of the genetic basis of adaptation in natural populations of nonmodel organisms. By screening large numbers of loci distributed throughout the genome, researchers are able to tease apart neutral (genome-wide) and adaptive (locus-specific or “outlier”) effects. This approach has been employed at multiple scales for investigating the genetic basis of adaptation, from continuous distributions along gradients of altitude (Bonin et al. 2006) and temperature (Jump et al. 2006), to phenotypically discrete ecotypes (Nosil et al. 2008) and subspecies (Nunes et al. 2011). When applied to populations along altitudinal gradients, where environmental conditions change rapidly over short distances, population genomics may be used to investigate expected temporal changes in selection pressures generated by climate change (Luikart et al. 2003).

**Figure 1:**
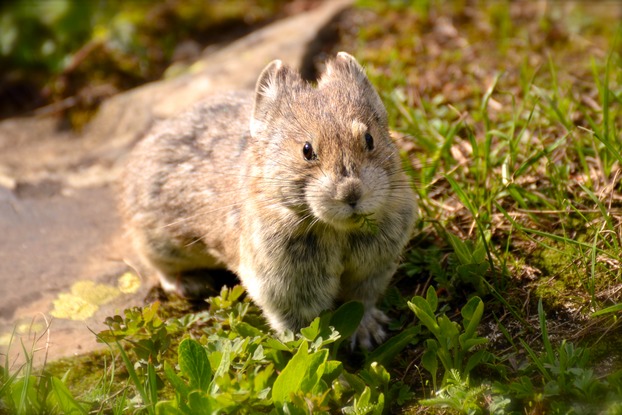
Photograph of an American pika (*Ochotona princeps*) foraging in a meadow close to some talus in the Bella Coola Valley British Columbia, Canada. Kindly contributed by Alison Henry.

The American pika (*Ochotona princeps*; Fig. 1) is a small lagomorph discontinuously distributed in mountainous areas throughout western North America. Pikas inhabit talus slopes in close proximity to meadows in which they forage. In recent years, *O. princeps* has been propelled to the position of a model mammalian species for studies of metapopulation dynamics, island biogeography, and source-sink dynamics (Clinchy et al. 2002). Given their limited tolerance to thermal stress, pikas have become a focal system for testing extinction dynamics in the face of climate change (Beever et al. 2011). In a study testing several alternative models, Beever et al. (2010) identified that climatic variables, especially mean summer temperature, (a measure of chronic heat stress), were the most influential factors driving pika extirpation. An *ad hoc* analysis showed maximum elevation, and the number of days with temperatures below −10°C (a measure of acute cold stress) was also strong predictors of pika extirpation (Ray et al. 2012). In that vein, pikas are considered harbingers of global warming, predicted by some to be the first mammalian species that may go extinct due to the direct effects of climate change (Smith et al. 2004).

In the present study, we used population and landscape genomic approaches to investigate the genetic basis of adaptation in *O. princeps* populations found along three elevations and one latitudinal gradient in the central Coast Mountains of British Columbia, Canada, ranging from sea level to 1500 m. Specifically, we addressed the following questions: (1) What proportion of the American pika genome is under positive selection in this system? and (2) What are the main environmental variables associated with adaptive population divergence in this species along independent elevation and longitudinal gradients?

## Materials and Methods

### Sampling design

**Figure 2:**
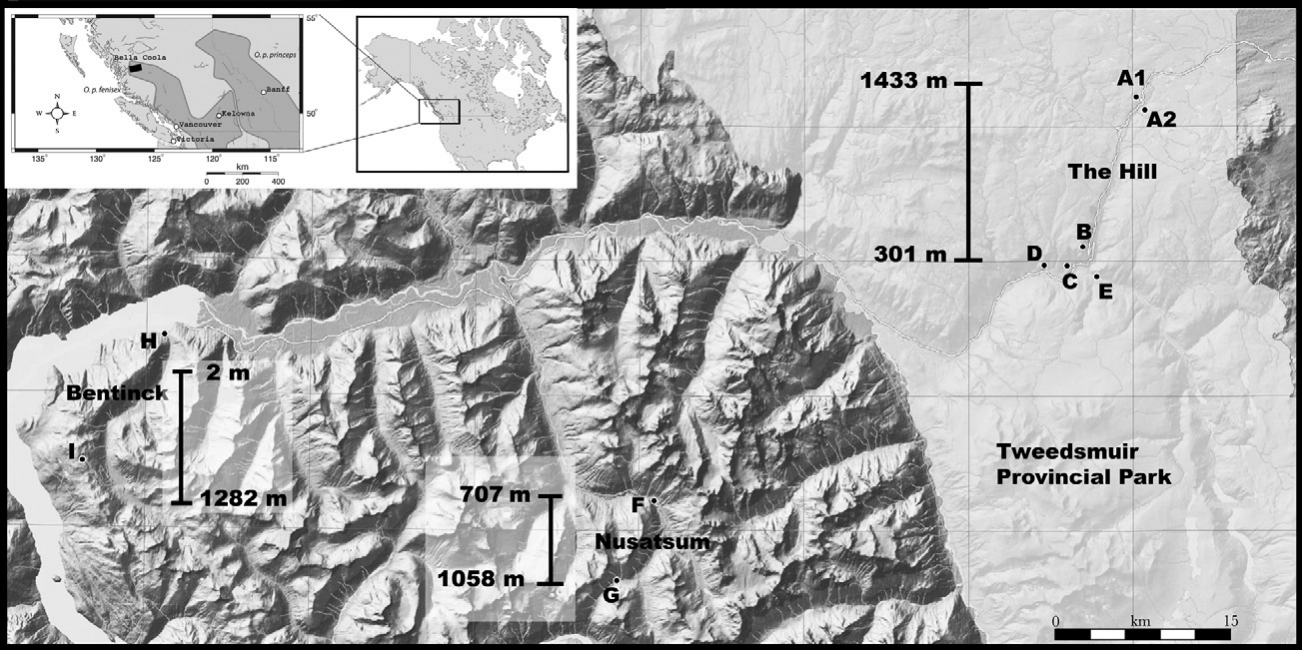
Map of the study area located in the Bella Coola Valley, British Columbia, Canada including the ten sampling sites located along three elevation gradients (lowest to highest elevations indicated): The Hill, Nusatsum and Bentinck from east to west. Insets indicate the location of the study area as well as the distribution of *O. princeps* in western North America.

This study was carried out in the Bella Coola Valley, BC, Canada (Fig. 2). This area was chosen for several reasons, including historical records of the occurrence of *O. princeps* from sea level to tree line. The valley runs from east to west and thus provides a longitudinal gradient from the interior to the coast, with marked differences in precipitation. American pikas were sampled from August 2008 to September 2010 at 10 sites along three elevation gradients (Hill, Nusatsum and Bentinck gradients) using recently developed noninvasive hair snares (Fig. 2; Henry and Russello 2011). The transects ranged from sea level to over 1400 m, representing strong temperature gradients, with differences of up to six degrees Celsius in mean summer temperatures from the bottom to the top of the Hill over a distance of only 16 km (Fig. 2; Henry et al. 2012a). At each site, hair snares were set up along travel routes (identified by observation of pika behavior) as well as at each detected haypile and latrine sites that were a minimum of 20 m apart.

A total of 288 georeferenced hair samples were previously collected and used for DNA extractions (Henry et al. 2012b). Multilocus microsatellite genotyping revealed the presence of 168 individuals (Henry et al. 2012b). At the Hill gradient, six populations were sampled (Table 1; Supplementary Data S1 and S2): two high-elevation sites, (A1 [*n* = 15]; A2 [*n* = 6]), one mid-elevation site (B [*n* = 17]), and three low elevation sites (C [*n* = 26]; D [*n* = 32]; E [*n* = 21]). At the Nusatsum gradient, one mid-elevation site (F [*n* = 10]) and one high-elevation site (G [*n* = 30]) were sampled. Lastly at the Bentinck gradient, one low elevation site, H (*n* = 5) and one high-elevation site, I (*n* = 6) were sampled. Each sampling site was defined as the entire area covered by talus, with the exception of site H, where sea level pikas inhabited the rocky interstice between forest and fjord. The surface area where sampling took place varied from 578 to 22,768 m^2^ (mean = 11,772, SD = 8690 m^2^).

**Table 1:** Site-specific information including site names, sample size (*N*), transect, geographical location, area (sq m), altitude (m), mean annual precipitation (MAP, mm), mean annual temperature (MAT, °C), precipitation as snow (PAS, mm), summer mean maximum temperature (*T*_max_, °C), and winter mean minimum temperature (*T*_min_, °C)

Site	*N*	Transect	Latitude	Longitude	Area (sq m)	Altitude (m)	MAP (mm)	MAT (°C)	PAS (mm)	*T*_max_ (°C)	*T*_min_ (°C)
A1	15	Hill	N52°18′36″	W125°29′47″	7867	1433	848	0.3	499	16.4	−14
A2	6	Hill	N52°18′26″	W125°29′34″	3971	1338	838	0.8	477	16.7	−13.5
B	17	Hill	N52°15′9″	W125°31′39″	16,030	793	706	2.7	359	19.4	−11.9
C	26	Hill	N52°14′56″	W125°32′14″	19,088	362	724	4.5	292	20.7	−9.6
D	32	Hill	N52°14′49″	W125°33′15″	17,849	301	775	4.7	297	20.7	−9
E	21	Hill	N52°14′39″	W125°31′14″	22,375	329	711	5	260	21.1	−8.9
F	10	Nusatsum	N52°9′37″	W126°11′29″	4734	707	2671	3.8	933	16.9	−7.4
G	30	Nusatsum	N52°7′46″	W126°13′4″	22,768	1058	2589	2.4	1,219	16.9	−10.3
H	5	Bentinck	N52°13′21″	W126°29′22″	578	2	2193	6.4	382	19.6	−4.1
I	6	Bentinck	N52°10′22″	W126°32′5″	2458	1282	2863	2.2	1374	16.8	−9.7

### AFLP genotyping and band scoring

We used amplified-fragment-length-polymorphism (AFLP)-based genomic scans to screen large numbers of molecular markers in the 168 sampled individuals. AFLPs produce high genomic coverage at a relatively low cost and constitute effective markers for genomic scans in nonmodel organisms without a reference genome (Bonin et al. 2007a). Approximately 50 ng of isolated DNA was digested sequentially using *Taq*I and *Eco*RI restriction endonucleases (New England Biolabs, Ipswitch, MA) following the protocol outlined in Bonin et al. (2005) with the following modifications: double-stranded adapters (Table S1) were prepared fresh each time the procedure was repeated and ligation took place at 16°C overnight in order to maximize the efficiency of the reaction. Following ligation, the reaction volume was diluted four times instead of 10 times. Preselective and selective amplifications were performed using a Veriti® thermal cycler (Applied Biosystems, Foster City, CA) in a 25 μL volume (Bonin et al. 2005). Initially, we used a representative sample of 16 individuals distributed throughout our study area and across elevation gradients to assess amplification success and polymorphism of all possible combinations of primers with three selective base pairs (Table S1). Based on results from the pilot analysis, twenty primer combinations that produced a large number of bands with high repeatability were retained and screened on the remainder of the sample. In order to quantify error rates and check repeatability of our protocol, 42 individuals (25% of total sample) were randomly selected as duplicates.

Cycling parameters for preselective and selective amplification were carried out according to Bonin et al. (2005) with the exception that we used KAPA *Taq* (KAPA Biosystems, Cape Town, South Africa) for the preselective amplification. Each *Eco*RI primer was fluorescently labeled (Table S1) to enable analysis on a capillary system. PCR products were multiloaded and run on an ABI 3130XL genetic analyser (Applied Biosystems) with GeneScan™ 600 LIZ® size standard.

AFLP profiles were called using a semi-automated approach in GeneMapper 4.0 (Applied Biosystems). First, we allowed GeneMapper to automatically generate bins of 1 base pair (bp) width between 50 bp and 600 bp. We manually checked all bins and removed those that overlapped. Fragments with a relative fluorescent unit (rfu) less than 50 were discarded as this threshold was used to represent instrument noise, and the peak heights were left as un-normalized. The output from GeneMapper (fragment size and peak heights) was then imported into scanAFLP 1.3 (Herrmann et al. 2010) for further processing. Marker selection proceeded by first discarding fragments with the following characteristics: (1) peaks lower than 200 rfu; (2) heights lower than 10% of the mean height of the maximum height frequency class; and/or (3) a coefficient of variation higher than one. In addition, markers that differed by more than one fragment among replicates were also discarded. The resulting binary matrix was handled with AFLPdat (Ehrich 2006) to produce files formatted for further analyses. A total of 1509 AFLP loci produced using 20 selective primer combinations were reliably scored, ranging in size from 50 to 473 base pairs. Each primer combination yielded on average 75 bands, with a mean error rate of 0.41% ranging from 0.2% to 0.9% (Table S2).

### Environmental data

Climatic data were calculated based on geographical location and elevation for each site using ClimateBC 3.1 (http://www.genetics.forestry.ubc.ca/cfcg/climate-models.html; Wang et al. 2006). This software downscales and interpolates PRISM 1961–1990 monthly normal data (2.5 × 2.5 arcmin) into 100 m × 100 m resolution and outputs a number of measured and derived variables. Initially, we targeted the 39 annual and seasonal environmental variables available through ClimateBC. In order to remove redundant information, we performed a principal component analysis (PCA) and calculated correlation coefficients between each pair of variables using the package ADE4 1.5-2 (Dray and Dufour 2007) in R version 3.0.1 (R Development Core Team 2012). Variables were considered redundant if they produced a correlation coefficient higher than 0.8 (Manel et al. 2010), in which case the variables that were deemed less biologically relevant (e.g., derived variables or variables that a priori do not affect the species) were removed from further analyses. We thus retained altitude (*ALT*), mean annual precipitation (MAP), mean annual temperature (MAT), precipitation as snow (PAS), summer mean maximum temperature (*T*_max_), and winter mean minimum temperature (*T*_min_) as the explanatory variables in tests of associations with allele frequency data (Table 1). While ALT is often considered as a covariate with other variables such as temperature and precipitation, it was not found to be redundant given our criteria, possibly due to the complex topography of the study area. ALT was also retained here as it has been identified as a strong predictor of pika population extirpation in previous modeling exercises (Beever et al. 2003, 2011). The other variables we retained were deemed biologically relevant based on previous work, including MAT and *T*_max_, as high summer temperatures have been shown to cause acute or chronic heat stress (Ray et al. 2012). Likewise, extreme cold temperatures in the winter have also been found to affect pika persistence (Beever et al. 2010), and snowpack was found to mitigate extreme colds by acting as an insulating blanket (Morrison and Hik 2007), thus we retained *T*_min_ and PAS. Lastly, we retained MAP, as drier sites are known to be less favorable to pikas, possibly due to an indirect effect on vegetation abundance (Ray et al. 2012).

### Detection of outlier loci

We used a set of alternative methods to identify loci potentially subject to selection in our system: (1) two frequentist *F*_st_ outlier detection methods relying on different simulation frameworks, (2) a Bayesian *F*_st_ outlier detection method, and (3) a spatial analysis method incorporating multiple linear regression models to correlate AFLP band frequencies with environmental variables. We chose to use the multiple methods available as each approach has its own assumptions and algorithm. We applied the frequentist method implemented in Mcheza (Antao and Beaumont 2011), which is based on the algorithm of Fdist (Beaumont and Nichols 1996). This software estimates allele frequencies using a Bayesian method and calculates *F*_st_ indices between predefined populations (10 sample sites). Coalescent simulations are performed under a finite island model to generate an *F*_st_ null distribution. Loci with unusually high or low *F*_st_ values contingent on their allele frequencies are considered outliers, and thus potentially under selection. We performed these analyses using 1,000,000 iterations and the most stringent settings including a 0.95 confidence interval (CI) and 5% false discovery rate (FDR) to guard against false positives. All other parameters used default values with θ = 0.1, β-a and β-b = 0.25 and a critical frequency of 0.99. Additionally, we used the neutral mean *F*_st_ and force mean *F*_st_ options. This undertakes an initial run and removes potential outliers in order to compute an unbiased neutral *F*_st_. The second frequentist method, also derived from Fdist (Beaumont and Nichols 1996) and implemented in Arlequin 3.5 (Excoffier and Lischer 2010), applies a hierarchical island model (Excoffier et al. 2009) that takes underlying population genetic structure into account in calculating *F*_st_. This method has been shown to substantially reduce false positive rates in hierarchically structured populations (Excoffier et al. 2009), such as that observed in our system (Henry et al. 2012b). We thus grouped populations belonging to the same elevation transect as suggested in this previous study and used a 95% CI to identify outliers. We applied 1,000,000 iterations and simulated 100 demes per group for 10 groups with minimum and maximum expected heterozygosities bounded between 0 and 1 under a pairwise difference model.

The second general approach we used for outlier detection was the Bayesian method implemented in BayeScan 2.01 (Foll and Gaggiotti 2008). This approach directly estimates the probability that each locus is subject to selection by teasing apart population-specific and locus-specific components of *F*_st_ coefficients using a logistic regression. The posterior probability of a given locus being under selection is assessed by defining two alternative models, one including the locus-specific effect and the other excluding it. Departure from neutrality is assumed when the locus-specific component is necessary to explain the observed pattern of diversity using a reversible jump Markov chain Monte Carlo (MCMC) algorithm that takes into account all loci at once, thus resolving the issue of multiple testing of a large number of loci. We ran chains of 1,000,000 iterations with a thinning of 10, resulting in 100,000 iterations considered following a burn-in of 50,000. As above, all other parameters were kept as default, including 20 pilot runs of 5000 iterations in length, prior odds for the neutral model of 10, and a uniform distribution of *F*_*is*_ between 0 and 1. As above, we opted for stringent settings, implementing a posterior probability of 0.76 and above and a 5% false discovery rate in order to identify outlier loci.

The third independent approach we used for outlier detection was the spatial analysis method, SAM (Joost et al. 2007, 2008; http://www.econogene.eu/software/sam/), that computes multiple univariate logistic regression models to test for associations between the frequency of AFLP bands and data from selected environmental variables. To ensure the robustness of the method, likelihood ratio (*G*) and Wald statistical tests are implemented to assess the significance of coefficients calculated by the logistic regression function. A model is considered significant only if the null hypothesis is rejected by both tests, after Bonferroni correction for multiple testing. For both tests, the null hypothesis is that the model with the examined variable does not explain the observed distribution better than a model with a constant only (Joost et al. 2008). We used a significance threshold corresponding to a 95% CI after Bonferroni correction.

After identifying loci that showed outlier behavior or significant association with the selected environmental variables using the approaches described above, we performed linear regression analysis using R. For each linear regression, the residuals were extracted and the assumption of linearity and normality was verified by plotting the distribution of residuals and corresponding Normal Quantile Plots. Homoscedasticity was verified by looking at each residual plot and using a Levene test for homogeneity of variances. Given the linearity, normality, and homoscedasticity of residuals, we calculated the adjusted *R*^2^ values (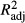) for each regression model separately and for each environmental variable. The 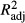 values were used as they provide an unbiased estimate of the explanatory power of each alternative model (Ohtani 2000). All above analyses were repeated four times, once at a regional scale using all the sampled populations representing a longitudinal gradient from the interior to the coast, and once across each of the three elevation gradients separately. In the latter cases, and with the exception of the Hill, the analyses using hierarchical population structure implemented in Arlequin 3.5 (Excoffier and Lischer 2010) and the SAM (Joost et al. 2007) analyses were omitted as it is not applicable to within-transect comparisons.

## Results

### Detection of outlier loci across a longitudinal gradient

We detected 58 outlier loci across all sampling sites along this longitudinal gradient (3.8% of the genomic scan; Table 2). The algorithm implemented in Mcheza (Antao and Beaumont 2011) identified 102 loci with *F*_st_ values significantly greater than that expected under a neutral model (95% significance level and a 5% FDR), indicative of positive or directional selection, 58 of which were also identified by at least one other approach (Table 2). Arlequin (Excoffier et al. 2009) identified 68 loci under selection (95% CI level), 51 of which were shared with Mcheza (Antao and Beaumont 2011). The Bayesian approach of BayeScan (Foll and Gaggiotti 2008) was the most conservative of all, detecting 12 outliers (posterior probability of 0.76% and 5% FDR), 8 of which were also identified by Arlequin (Excoffier et al. 2009), while all 12 were also detected by Mcheza (Antao and Beaumont 2011). Lastly, the SAM (Joost et al. 2008) identified 11 loci potentially associated with four of the six environmental variables under study (MAP, PAS, *T*_max_*,* and *T*_min_*;* 95% confidence level incorporating Bonferroni corrections with a confidence threshold of 5.52 × 10^−6^), all of which were also identified as outliers by one or more of the *F*_st_-based methods (Table 2).

**Table 2:** List of outlier loci detected by four methods across our entire sample

Transect	Comparison	Marker^1^	Mcheza^2^	Bayescan^3^	Arlequin^4^	SAM^5^	Linear (R_adj_^2^, *F*, *P*-value)^6^
Longitudinal	Algorithm	E31T37_100	0.991	0.779	0.004	MAP^7^; PAS^7^	(0.65, 17.8, 0.003); (*0.25, 4.1, 0.08*)
Longitudinal	Algorithm	E31T37_104	0.964	0.836	–	PAS^8^; MAP^7^; *T*_min_^8^	(0.36, 6.13, 0.04); (0.84, 47.27, 0.0001); (0.39, 6.63, 0.03)
Longitudinal	Algorithm	E31T37_105	0.972	–	0.015	–	
Longitudinal	Algorithm	E31T37_51	0.998	–	0.024	–	
Longitudinal	Algorithm	E31T37_99	–	0.840	–	MAP^7^; *T*_min_^8^	(0.65, 17.8,0.003); (0.36, 6.14, 0.04)
Longitudinal	Algorithm	E31T39_108	0.999	–	0.002	–	
Longitudinal	Algorithm	E31T39_53	0.987	0.901	–	MAP^7^; PAS^7^	(0.56, 12.3, 0.008); (*0.32, 3.7, 0.09*)
Longitudinal	Algorithm	E31T39_56	0.999	–	0.000	–	
Longitudinal	Algorithm	E31T39_62	0.957	–	0.013	MAP^7^; PAS^8^	(0.71, 23, 0.001); (0.44, 7.9, 0.02)
Longitudinal	Algorithm	E31T39_84	0.965	0.927	0.023	MAP^8^; PAS^8^	(0.46, 8.8, 0.02); (*0.35, 4.4, 0.07*)
Longitudinal	Algorithm	E31T39_88	0.999	–	0.002	–	
Longitudinal	Algorithm	E31T43_53	0.985	–	0.029	–	
Longitudinal	Algorithm	E31T43_82	0.999	0.887	0.004	–	
Longitudinal	Algorithm	E32T35_112	0.971	–	0.014	–	
Longitudinal	Algorithm	E32T35_53	0.985	–	0.030	–	
Longitudinal	Algorithm	E33T32_104	0.965	–	0.030	–	
Longitudinal	Algorithm	E33T32_93	0.999	–	0.020	–	
Longitudinal	Algorithm	E33T37_103	–	–	0.041	MAP^7^; *T*_min_^8^	(0.66, 18.2, 0.003); (0.4, 6.9, 0.03)
Longitudinal	Algorithm	E33T37_105	0.987	–	0.019	–	
Longitudinal	Algorithm	E33T39_54	0.999	–	0.001	–	
Longitudinal	Algorithm	E33T39_56	0.966	–	0.009	–	
Longitudinal	Algorithm	E33T39_58	0.999	0.926	0.032		
Longitudinal	Algorithm	E33T39_59	0.999	–	0.019	–	
Longitudinal	Algorithm	E33T39_86	0.995	0.923	0.005	–	
Longitudinal	Algorithm	E33T39_89	0.969	0.916	0.015	MAP^8^; PAS^8^; *T*_max_^8^	(0.44, 8.1, 0.02); (0.33, 5.5, 0.02); (0.43, 7.7, 0.02)
Longitudinal	Algorithm	E33T39_91^9^	0.992	0.974	0.013	*T*_max_^7^; MAP^8^	(0.41, 7.3, 0.03); (*0.24, 3.9, 0.08*)
Hill	Algorithm	E33T39_91^9^	0.985	–	–	*T*_min_^8^	(0.72, 10.4, 0.03)
Longitudinal	Algorithm	E34T38_83	0.994	–	0.020	–	
Longitudinal	Algorithm	E34T38_92	0.982	–	0.010	–	
Bentinck	Transect	E34T44_57^9^	0.989	–	NA	NA	NA
Hill	Transect	E34T44_57^9^	0.970	–	–	–	
Hill	Transect	E34T45_103^9^	0.995	–	–	–	
Nusatsum	Transect	E34T45_103^9^	0.995	–	NA	NA	NA
Longitudinal	Algorithm	E34T45_122	0.985	0.848	–	–	
Hill	Transect	E34T45_144^9^	0.999	–	–	–	
Nusatsum	Transect	E34T45_144^9^	0.955	–	NA	NA	NA
Longitudinal	Algorithm	E34T45_51^9^	0.999	–	0.023	–	
Hill	Transect	E34T45_51^9^	0.965	–	–	*T*_min_^8^	(0.54, 8.3, 0.04)
Nusatsum	Transect	E34T45_51^9^	0.988	–	NA	NA	NA
Longitudinal	Algorithm	E34T45_56	0.995	–	0.038	–	
Longitudinal	Algorithm	E34T45_86	0.994	–	0.026	–	
Longitudinal	Algorithm	E38T32_126	0.998	–	0.018	–	
Longitudinal	Algorithm	E38T32_136^9^	0.963	–	0.024	–	
Hill	Algorithm	E38T32_136^9^	0.951	–	–	MAP^7^; *T*_max_^7^	(0.82, 24, 0.008); (0.82, 23, 0.009)
Longitudinal	Algorithm	E38T32_160	0.963	–	0.020	–	
Longitudinal	Algorithm	E38T32_80	0.963	–	0.034	–	
Longitudinal	Algorithm	E38T32_91	0.977	–	0.011	–	
Hill	Algorithm/Transect	E38T37_105^9^	0.973	0.790	–	MAP^8^; *T*_max_^8^	(0.6, 8.4, 0.04); (0.58, 7.9, 0.04)
Nusatsum	Transect	E38T37_105^9^	0.999	–	NA	NA	NA
Longitudinal	Algorithm	E38T37_155	0.999	–	0.009	–	
Longitudinal	Algorithm	E38T37_52	0.990	–	0.040	–	
Longitudinal	Algorithm	E38T37_53	0.996	–	0.033	–	
Hill	Algorithm	E38T37_60	0.998	–	–	*T*_max_^8^	(0.71, 13.3, 0.02)
Longitudinal	Algorithm	E38T37_83	0.985	–	0.027		
Longitudinal	Algorithm	E43T35_57	0.999	–	0.005	–	
Longitudinal	Algorithm	E43T35_61	0.954	–	0.037	–	
Longitudinal	Algorithm	E43T35_68	0.999	–	0.014	–	
Longitudinal	Algorithm	E43T37_213	0.999	–	0.000	–	
Longitudinal	Algorithm	E43T37_215^9^	0.999	0.999	0.000		
Hill	Transect	E43T37_215^9^	0.984	–	–	–	
Nusatsum	Transect	E43T37_215^9^	0.988	–	NA	NA	NA
Longitudinal	Algorithm	E43T37_51	0.997	–	0.012	–	
Longitudinal	Algorithm	E43T37_53	0.990	–	0.045	–	
Longitudinal	Algorithm	E43T43_104	–	–	0.044	MAP^8^; PAS^7^	(0.37, 6.2, 0.04); (0.7, 22.3, 0.001)
Hill	Algorithm	E43T43_80	0.999	–	–	PAS^8^; *T*_max_^8^	(0.76, 16.9, 0.01); (0.79, 20.3, 0.01)
Longitudinal	Algorithm	E43T44_107	0.953	–	0.025	–	
Longitudinal	Algorithm	E43T44_87	0.997	–	0.013	–	
Longitudinal	Algorithm	E43T44_88	0.972	–	0.022	–	
Nusatsum	Algorithm	E44T38_115	0.999	0.810	NA	NA	NA
Longitudinal	Algorithm	E44T38_124	0.968	–	0.025	–	
Longitudinal	Algorithm	E44T38_71	0.971	–	0.047	–	
Longitudinal	Algorithm	E44T38_72	0.954	–	0.016	–	
Longitudinal	Algorithm	E44T38_87	0.991	–	0.018	–	
Longitudinal	Algorithm	E44T44_104	0.999	–	0.028	–	
Hill	Transect	E46T38_125^9^	0.999	–	–	–	
Nusatsum	Transect	E46T38_125^9^	0.990	–	NA	NA	NA
Bentinck	Algorithm/Transect	E46T38_65^9^	0.998	0.770	NA	NA	NA
Hill	Transect	E46T38_65^9^	0.953	–	–	–	
Nusatsum	Transect	E46T38_65^9^	0.967	–	NA	NA	NA
Longitudinal	Algorithm	E46T45_76^9^	0.964	–	–	*T*_max_^7^	(0.67, 19.1, 0.002)
Hill	Algorithm	E46T45_76^9^	0.957	–	–	*T*_max_^7^	(0.74, 15.1, 0.02)

1 Outliers detected by either more than one algorithm within a transect (Algorithm) or detected independently in more than one elevation transect (Transect).

2 For Mcheza, 95% significance level and 5% false discovery rates were used.

3 For Bayescan, a posterior probability above 0.76 indicated a strong outlier with a 5% FDR.

4 For Arlequin, a 95% CI level was used to identify an outlier.

5 For the SAM, the environmental variables significantly correlated at a 95%and 99% CI and after Bonferroni corrections (confidence threshold at 5.52 × 10^−6^ and 1.1 × 10^−6^) are indicated. Climatic variable abbreviations are as follows: MAP, mean annual precipitation; PAS, precipitation as snow; *T*_max_, summer maximum temperature; *T*_min_, winter minimum temperature.

6 Values in italic indicate relationships that were marginally significant using linear regression.

7 Statistically significant at *P* < 0.01, corresponding to a confidence threshold after Bonferroni corrections of 1.1 × 10^−6^.

8 Statistically significant at *P* < 0.05, corresponding to a confidence threshold after Bonferroni corrections of 5.52 × 10^−6^.

9 Indicates outliers loci that were detected among multiple individual transects.

**Figure 3:**
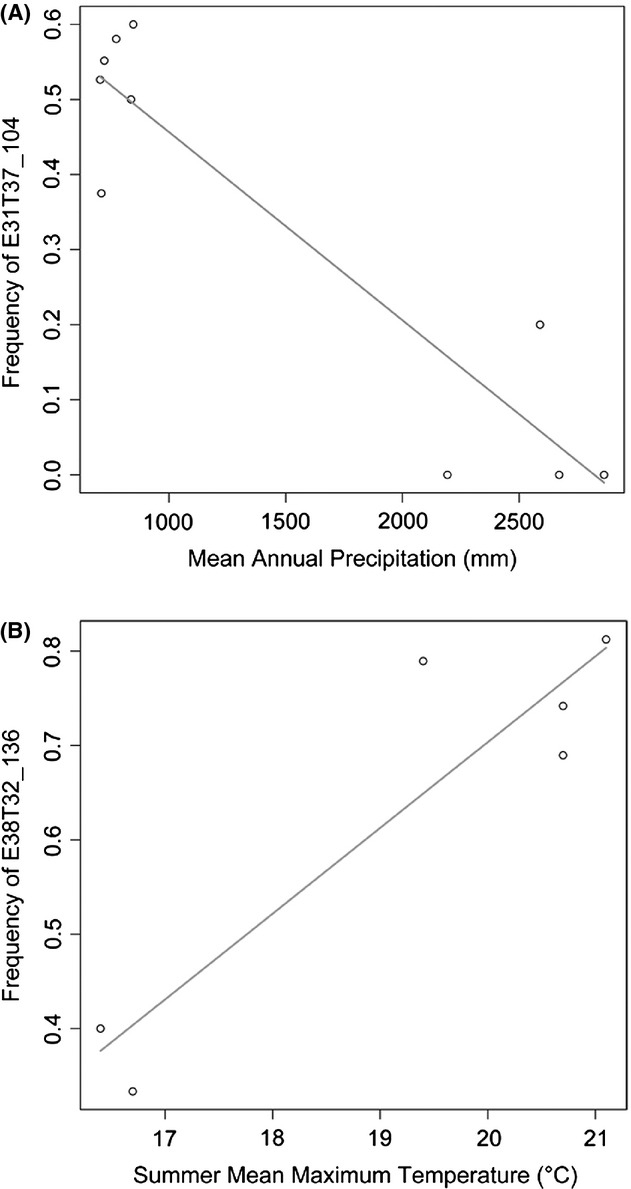
(A) Linear regression of the frequency of E31T37_104 against mean annual precipitation (MAP), depicting a significant negative relationship (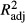 = 0.84, *F*-test, *F* = 47.27, DF = 8, *P* = 0.0001) across the longitudinal gradient from coast to interior. (B) Linear regression of the frequency of E38T32_136 against summer mean maximum temperature (*T*_max_), depicting a significant negative relationship (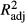 = 0.82, *F*-test, *F* = 23, DF = 6, *P* = 0.009) across the Hill elevation gradient. Points indicate sampling locations.

Three outliers (E31T39_84, E33T39_89, and E33T39_91) were identified by all methods and were found to be associated with mean annual precipitation (MAP), precipitation as snow (PAS) and mean temperature of the warmest months (*T*_max_). After performing linear regression analysis on each of the 58 outliers with each of the six environmental variables separately, ten loci displayed strong and significant associations with MAP, PAS, *T*_min_*,* and/or *T*_max_ (Table 2; Fig. 3A).

### Detection of outlier loci across elevation gradients

The above analyses were repeated separately for each transect (Fig. 2) in order to evaluate the potential selection pressures associated with elevation. Along the Hill transect, we identified a total of six outliers detected by multiple algorithms (0.4% of the genomic scan; Table 2), each of which also exhibited a significant association with at least one environmental variable based on the SAM (Fig. 3B; Joost et al. 2008). Five outlier loci detected along the Hill transect overlapped with those found in the regional scale analysis (Table 2). At both the Nusatsum and Bentinck transects, only a single, nonoverlapping locus was identified by multiple algorithms (Table 2). In both cases, the SAM (Joost et al. 2008) algorithm reached its iteration limit and produced no results, possibly caused by the fact that these transects contained only two populations each.

Interestingly, Mcheza (Antao and Beaumont 2011) identified eight loci that were detected independently as outliers in two or more transects (Table 2). One locus was detected independently in all three transects (E46T38_65) and corresponded to the only locus detected by multiple algorithms in Bentinck (Table 2). Three loci independently detected in the Hill and Nusastum transects were also identified in the longitudinal analysis (E34T45_51, E43T37_215; Table 2) or by multiple algorithms in the Hill transect alone (E38T37_105; Table 2). Two of these eight loci (E38T37_105 and E24T45_51) also showed a significant association with at least one environmental variable along the Hill transect.

## Discussion

While climate change is regarded as a potential threat to species persistence, we have relatively little understanding of the genetic basis underlying biotic responses to environmental changes. This information will likely become increasingly important for the design and implementation of conservation strategies that explicitly take environmental changes into consideration, potentially informing conservation unit delimitation (Funk et al. 2012) and direct intervention in the form of assisted migration (Allendorf et al. 2010). In order to meaningfully inform such strategies, it is important to better understand the extent and distribution of adaptive genetic variation, as well as the underlying drivers of divergence. In this study, we used AFLP-based genomic scans to begin to fill this knowledge gap for American pika, a sentinel mammalian species of growing conservation concern.

### The extent and distribution of outlier loci

We identified 3.8% of loci analyzed as displaying evidence for positive selection or significant association with an environmental variable at the regional scale. This percentage falls below the reported values for AFLP-based genomic scans using Dfdist (5–10%) (Nosil et al. 2009). A possible explanation for the lower percentage of outliers detected in our study is the fact that it was conducted within a subspecies of American pika (*O. princeps fenisex*) and at a finer scale in comparison with previous work (but see Parisod and Joost 2010). Indeed, other studies have used populations that were separated by several hundred kilometers (Nunes et al. 2011), or at both continental and regional scales (Manel et al. 2010). The only comparable study in vertebrates is that of Bonin et al. (2006) that focused on common frog populations sampled along an altitudinal gradient and recovered a similar percentage of outliers as those reported here.

It can be challenging, however, to compare percent outliers between studies, given that no convention has yet been established regarding the type and number of algorithms used and their associated significance criteria (but see De Mita et al. 2013) Here, we implemented a conservative approach to outlier locus detection, employing both *F*_st_-based and environment–allele association approaches, and only identifying outliers as loci detected by at least two independent tests (Perez-Figueroa et al. 2010) or by a single test across multiple environmental transects. The cost associated with this approach, however, may have led to an inflated type II error, effectively underestimating the proportion of outlier loci in our sample compared with other studies. Nonetheless, cross-validation appears to be an effective strategy for increasing confidence in identified outliers. For example, the algorithm implemented in Arlequin (Excoffier et al. 2009) that takes into account hierarchical population structure in the detection of outlier loci was largely consistent with Mcheza (Antao and Beaumont 2011), which does not account for population structure and implements a finite island model. In this case, only four loci were identified as outliers by the latter, but not the former. Yet these four loci were not regarded as false positives as they were also detected independently by the SAM (Joost et al. 2008).

While differences in sampling design for each transect precluded the use of all the analyses described above, our experimental design still enabled us to make meaningful comparisons with regards to differences in outlier detection across multiple environmental gradients. First, a larger proportion of outlier loci were detected along the latitudinal gradient as compared with that found in altitudinal gradients. Reasons for this discrepancy include differences in the numbers of populations tested (10 for latitudinal and 6, 2, and 2 for altitudinal gradients) and spatial scale (70 km for latitudinal and approximately 10 km for altitudinal gradients). Second, each elevation gradient was characterized by its own set of outlier loci, which may be the result of divergent population histories within each transect. The populations from the Hill displayed the largest amount of outliers detected by multiple independent approaches, while Nusatsum and Bentinck yielded eight and two outliers each. This discrepancy between proportion of outliers detected may be due to differences in sampling design (2 vs. 6 populations) as well as differences in sample size. Interestingly, eight loci were detected independently as outliers in two or more transects (Table 2). One locus was detected independently in all three transects (E46T38_65) and corresponded to the only locus detected by multiple algorithms in Bentinck (Table 2). Three loci independently detected in the Hill and Nusastum transects were also identified in the longitudinal analysis (E34T45_51, E43T37_215; Table 2) or by multiple algorithms in the Hill transect alone (E38T37_105; Table 2). Two of these loci (E38T37_105 and E24T45_51) also showed a significant association with at least one environmental variable along the Hill transect. As these loci were generally not detected in the longitudinal transect (except E43T37_215), they may signify cases of convergent evolution across the multiple elevational transects, representing promising targets for further study.

### Environmental drivers of adaptive population divergence

Use of environmental–allele association approaches to outlier detection offer the added benefit of pinpointing putative mechanisms underlying divergence. Here, the SAM (Joost et al. 2008) identified loci that were significantly correlated with several environmental variables along the longitudinal gradient. Of eleven loci that showed significant association, 10 were correlated with MAP, seven with PAS, and three with *T*_max_ and *T*_min_. The amount of precipitation is thus considered as the main driver of population adaptive divergence across this 70 km longitudinal gradient ranging from the wet coast to the drier interior Coast Mountains. For example, locus E31T37_104 displayed a negative association with mean annual precipitation (Fig. 3A) and was entirely absent from populations experiencing high amounts of precipitation. While a direct impact of precipitation on pikas may be difficult to infer, we hypothesize that taken as a proxy for the quality of snowpack, MAP and PAS could potentially explain variation for traits related to cold tolerance, and could shed light on populations better adapted to acute cold stresses.

Within the Hill transect, seven loci were found to be correlated with environmental variables. *T*_max_ was associated with four outliers, followed by *T*_min_ (three loci), MAP (two loci), and PAS (one locus). Along this elevational gradient, the mean maximum temperature of the summer months was found to be the main driver of adaptive population divergence. When analyzed separately, two loci (E43T43_80 and E38T32_136) showed significant and strong correlation with *T*_max_*,* PAS, and MAP. Interestingly, E43T43_80 displayed a negative relationship with *T*_max_, and E33T39_91 displayed a positive relationship with *T*_min_, representing promising candidates for further studying a potential association with adaptation to cold tolerance in pikas. In contrast, E38T32_136 showed the opposite trend (Fig. 3B), potentially representing a variant associated with adaptation to warmer environmental conditions. In a conservation context, these loci could be screened in other populations and their relative frequencies used to calculate a population adaptive index, which may help prioritize populations for conservation action with regard to anticipated climate changes (Bonin et al. 2007b; Sgrò et al. 2011). This information could be then be used to guide-assisted migration efforts.

Although AFLP-based genomic scans are a repeatable and cost-effective way to screen the genome of nonmodel organisms, the anonymity of these markers precludes the identification of genes responsible for the observed adaptive divergence. Additional work is necessary to isolate, clone, and sequence these fragments with the aim to identify candidate genes associated with local adaptation, although this approach has not proven fruitful in our system and has been a challenge in others (Nunes et al. 2012). Thus, the present genomic scan represents a first broad attempt at characterizing adaptive population divergence in *O. princeps*. Follow-up studies will target recently developed SNP markers (Lemay et al. 2013). These efforts will bridge the gap between the anonymous candidates identified here and the chromosomal location and biochemical pathways associated with local adaptation in this system.

## Conclusion

The present study represents one of the first parallel uses of population and landscape genomic approaches to detect candidate loci under positive selection in a wildlife species potentially threatened by climate change. We illustrate the complementary nature of both approaches in identifying candidate loci responsible for local adaptation. Because these algorithms are prone to false positives, we opted to use multiple approaches and only identified outliers as loci detected by at least two independent tests or independent elevation transects. Based on this rationale, 4.5% of the genomic scan was detected as outlier loci, which lies at the low level of what has been reported in previous studies. Two main environmental variables, mean annual precipitation and summer mean maximum temperatures, were identified as forces associated with adaptive divergence in this system. Further exploration of candidate loci that demonstrated strong correlation with these environmental variables may represent important targets for predicting pika responses to climate change and informing novel approaches to wildlife conservation in a changing world.

## Data archiving

Both genetic and ecological data sets generated in the study are available on DRYAD (http://www.datadryad.com).

## Supporting Information

**Table S1.** AFLP adapters, primers and primer combinations used.

**Table S2.** Error rate for each of 20 primer combinations, corresponding to three different stages of the quality control procedure implemented in our study.
