# Supplementary material for: Adaptive divergence along environmental gradients in a climate-change-sensitive mammal
Source: Ecol Evol. 2013 Sep 16;3(11):3906–17. doi: 10.1002/ece3.776 (PMC3810883; doi:10.1002/ece3.776)
Supplement: Supplementary file 3 [file ece30003-3906-SD3.docx]

AFLP adapters, primers and primer combinations used.

Error rate for each of 20 primer combinations, corresponding to three different stages of the quality control procedure implemented in our study.
